# Epidemiological aspects of leprosy in Juazeiro-BA, from 2002 to 2012*

**DOI:** 10.1590/abd1806-4841.201533963

**Published:** 2015

**Authors:** Maria Eduarda Gomes da Cruz Silva, Carlos Dornels Freire de Souza, Susanne Pinheiro Costa e Silva, Flávia Monteiro da Costa, Rodrigo Feliciano do Carmo

**Affiliations:** 1 Universidade Federal do Vale do São Francisco (Univasf) - Petrolina (PE), Brazil; 2 Prefeitura Municipal de Juazeiro - Juazeiro (BA), Brazil; 3 Secretaria Municipal de Saúde de Petrolina - Petrolina (PE), Brazil

**Keywords:** Leprosy, Epidemiology, *Mycobacterium leprae*, Public health

## Abstract

**BACKGROUND:**

Leprosy is an infectious disease caused by *Mycobacterium leprae*, able to infect large numbers of people. This work is relevant to Juazeiro-BA, a hyper-endemic area for leprosy, since unravel the behavior of the disease in the area, may suggest the decision making for sectors of surveillance, establishing strategies, organizing and evaluating programs and services.

**OBJECTIVES:**

To analyze the epidemiology of leprosy in Juazeiro-BA, from 2002 to 2012.

**METHODS:**

A descriptive, cross-sectional study was conducted based in data of the Diseases Notification System, assigned by the service of Epidemiology from Juazeiro-BA, between 2002 and 2012.

**RESULTS:**

1,916 new cases of leprosy were detected between 2002 and 2012, of which 921 (48.07%) represented male sex, 995 (51.93%) female, and there was a reduction in the incidence rate of leprosy per 100,000 inhabitants. Most carriers were brown individuals, with low levels of education, living in the urban area, being more prevalent in the economically active age group. Through statistical analysis we found that there are more chances of developing sequelae among men, and multibacillary individuals older than 45 years.

**CONCLUSIONS:**

The work serves to direct efforts to control this disease, and highlights the importance of active search for new cases to achieve an early diagnosis, reducing the number of sequels and allowing breaking the chain of disease transmission.

## INTRODUCTION

Leprosy is a chronic infectious disease, with slow evolution, curable, caused by Hansen's bacillus - *Mycobacterium leprae*, which is able to infect a large number of people.^1,2^ The high disabling potential of leprosy is directly related to the penetration ability of the bacillus in the nerve cell and its immunogenic power, causing after-effects in individuals.^3^

This disease manifests itself mainly through dermatological-neurological signs and symptoms such as lesions in skin and peripheral nerves, especially in the eyes, hands and feet, being the involvement of the peripheral nerves the main feature of the disease, giving it great potential to cause physical disabilities that may even develop into deformities. These disabilities and deformities may result in other problems such as decreased ability to work, limitation of social life and psychological problems.^4,5^

In Brazil, leprosy still stands out as a major public health problem because it is the second country with the highest number of new reported cases, accounting for almost 93% of cases in the Americas.^1,2^ The country presents a high incidence rate (19.64/ 100,000 inhabitants), and a mean prevalence rate of 1.54/ 10,000 inhabitants.^6,7^ Although there is a decrease in the prevalence of leprosy in Brazil, the incidence rate has not reached an effective reduction.^2^

Between 2001 and 2008, 370,162 new cases of the disease were reported in Brazil. In 2011, 29,690 cases of leprosy were identified as active records in the country, thus Brazil is considered a country of average prevalence. The Northeast region presented 12,575 cases as active records, with a prevalence of 2.35 cases per 10,000 inhabitants, while leprosy incidence rate in Salvador, Bahia's capital, was 14.80 new cases per 100,000 inhabitants in 2010.^7,8^

Apart from the interregional heterogeneity of leprosy, there are also intermunicipal differences in detecting the disease, considered high in Brazil.^2^

The city of Juazeiro is located on the right bank of São Francisco River, in the far north of Bahia, in the submedium São Francisco region, with a population estimated at 197,965 inhabitants, according to Instituto Brasileiro de Geografia e Estatística - IBGE (2010). The city borders the state of Pernambuco, being connected to the city of Petrolina by the bridge Presidente Dutra, and it is 500 km far from Salvador.

Juazeiro is considered an hyperendemic city to leprosy, with a incidence rate of 103.6 cases per 100,000 inhabitants in 2010. Regarding children under 15 years old, the incidence rate in the same year was 41.89 cases per 100,000 inhabitants.^9^

Given the high endemicity of the disease in the city, this study aimed to determine the epidemiological aspects of leprosy in the city of Juazeiro, between 2002 and 2012. This study will contribute giving subsidies for actions against this municipal hazard and by creating strategies in public health aiming the control of leprosy.

## MATERIALS AND METHODS

This is an epidemiological descriptive study, with cross-sectional and retrospective character, which was held in Juazeiro-BA.

The study population consisted of all inhabitants of the city who were new diagnosed cases of leprosy from 2002 to 2012, registered in the Diseases Notification System (Sistema de Informação de Agravos de Notificação - SINAN), regardless of the clinical form, being excluded misdiagnosed cases. All reported cases were from primary care network and diagnosed by a dermatologist.

Calculation of leprosy incidence rate per 100,000 inhabitants was carried out using the formula provided by the Ministry of Health, Decree No. 3,125, of October 7th, 2010: Annual coefficient of new leprosy cases per 100,000 inhabitants: new cases per total population x 100,000. The necessary population data were provided by the Departamento de Informática do Sistema Único de Saúde (DATASUS).

The preparation of charts and tables were performed in Microsoft Excel^®^ version 2013. The SPSS program, version 17.0 (SPSS, Inc., Chicago, IL) was used for statistical analysis. Data were presented in relative and absolute frequency. Categorical variables were compared using the chi-square test. Binary logistic regression was performed to identify predictors related to the rate of disability. Results were presented using odds ratio (OR) with confidence interval (CI) of 95%. Differences were considered significant when *P* <0.05.

This study was approved by the Committee of Ethics and Deontology in Studies and Research of Universidade Federal do Vale do São Francisco, and is registered under the protocol number 0010/101213 CEDEP/ UNIVASF.

## RESULTS

Regarding data analysis and processing, it was possible to trace the epidemiological clinical profile of leprosy in the city of Juazeiro in the proposed period, 1,916 new cases were detected between 2002 and 2012. Among them, 921 (48.07%) were men and 995 (51.93%) were women.

**Figure 1:**
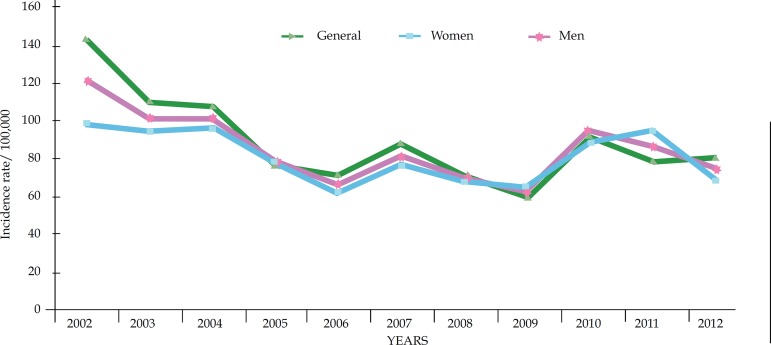
General incidence rate, men and women, per 100,000 inhabitants, in the city of Juazeiro-BA, between 2002 and 2012

Figure 1 shows that there was a decrease of the overall incidence rate over the years in both sexes. The highest value of the incidence rate was observed in 2002 (121.60/100,000 inhabitants). This rate was higher among women (143.00/100,000 inhabitants) compared to men (98.79/100,000 inhabitants). In 2012, the overall incidence rate decreased to 74.94/100,000 inhabitants, keeping the difference between sexes, men: 68.81/100,000 inhabitants and women 80.83/100,000 inhabitants.

Table 1 presents the frequency by age according to sex, between 2002 and 2012, showing that men in the age group of 31-45 years are the most affected, 276 cases (29.97%), while women are most affected in the age group 46-60 years, with 261 cases (26.23%).

**Table 1:** Socio-demographic characteristics of patients with leprosy in the city of Juazeiro-BA, by gender, between 2002 and 2012

Characteristics	Men		Women	
	n=921 %		n=995 %	
**Age (Years)**				
≤ 15	80	8.69	94	9.45
16-30	198	21.50	232	23.32
31-45	276	29.97	250	25.12
46-60	185	20.08	261	26.23
≥ 61	182	19.76	158	15.88
**Race**				
White	139	15.09	160	16.08
Black	154	16.72	135	13.57
Yellow	6	0.65	10	1.01
Brown	554	60.15	586	58.89
Indian	2	0.22	3	0.30
Not informed	10	1.09	13	1.31
Blank	56	6.08	88	8.84
**Education**				
Illiterate	134	14,55	116	11,66
Incomplete elementary school	505	54,83	564	56,68
Complete elementary school	32	3,47	24	2,41
Incomplete high school	96	10,42	124	12,46
Complete high school	57	6,19	55	5,53
Incomplete higher education	2	0,22	3	0,30
Complete higher education	35	3,80	38	3,82
Not informed	38	4,13	43	4,32
Blank	12	1,30	17	1,71
Not applicable	10	1,09	11	1,11
**Home location**				
Urban	820	89,03	884	88,85
Countryside	94	10,21	106	10,65
Periurban	5	0,54	2	0,20
Blank	2	0,22	3	0,30

Also regarding the distribution of leprosy by age group, we highlight the prevalence of cases in children under 15 years: 80 cases (8.69%) among boys and 94 cases (9.45%) among girls.

Regarding race, for both sexes, brown individuals were predominant, with 554 (60.15%) cases among men and 586 (58.89%) among women. Among men, the second highest frequency occurred in black individuals - 154 cases (16.72%), while in women, the second highest frequency occurred in white individuals - 160 cases (16.08%).

The level of education among the most frequent carriers of leprosy was incomplete elementary school, accounting for 505 cases (54.83%) among men and 564 cases (56.68%) among women. The second most frequent level of education was illiterate among men - 134 cases (14.55%) - and incomplete high school among women, - 124 cases (12.46%). Leprosy affected less frequently individuals with incomplete higher education for both sexes.

Regarding place of residence, urban area presented the highest frequency of carriers: 820 men (89.03%) and 884 women (88.85%). In the countryside, on the other hand, there was a low number of cases - 94 (10.21%) men and 106 (10.65%) women.

This study sought predictors of development of physical disability. Table 2 shows data from multivariate analysis between studied variables and the presence of some disability at diagnosis. After analyzing the binary logistic regression, variables identified as good predictors were: age, 213 (58.20%) individuals over 45 years presented disability grade I or II; men, with 224 cases (61.20%); and multibacillary operational classification, which presented a total of 286 cases (78.14%) with disabilities. These patients were 6 times more likely (OR = 6.31, 95% CI: 4.75 to 8.37) to develop some physical disability. All of these factors were statistically significant (p <0.05).

**Table 2:** Logistic regression analysis to determine predictors related to the rate of disability in patients with leprosy in the city of Juazeiro-BA between 2002 and 2012

Variables	Disability rate		p-value	OR*	(95% CI)
	I or II	None			
**Gender**					
Male	224	639	0.001	1.55	1.19-2.02
	(61.20%)	(44.94%)			
					
Female	142	639	783		
	(38.80%)	(55.06%)			
					
**Age (Years)**					
> 45	213	507	0.000	2.20	1.70-2.86
	(58.20%)	(35.65%)			
					
≤ 45	153	915			
	(41.80%)	(64.35%)			
					
**Operational classification**					
Multibacillary	286	461	0.000	6.31	4.75-8.37
	(78.14%)	(32.42%)			
					
Paucibacillary	80	961			
	(21.86%)	(67.58%)			

* OR: Odds Ratio.

In Table 3 we can observe that the frequency of paucibacillary individuals was high in every year. The highest frequency was in 2002 (130 cases) and the lowest in 2008 (81 cases). Multibacillary operational classification was also more frequent in 2002 (94 cases), but, on the other hand, the lowest frequency was observed in 2012 (58 cases). The highest difference found between the proportion of paucibacillary and multibacillary individuals occurred in 2006 (ratio of 1.70).

**Table 3:** Operational classification of patients with leprosy in the city of Juazeiro-BA, between 2002 and 2012

**Year**	**Classification paucibacillary**	**Multibacillary**	**Total**	PB/MB Ratio*
2002	130 (58.03%)	94 (41.97%)	224	1.38
2003	111 (59.68%)	75 (40.32%)	186	1.48
2004	105 (55.26%)	85 (44.74%)	190	1.23
2005	88 (57.52%)	65 (42.48%)	153	1.35
2006	87 (63.04%)	51 (36.96%)	138	1.70
2007	101 (58.05%)	73 (41.95%)	174	1.38
2008	81 (51.60%)	76 (48.40%)	157	1.06
2009	85 (56.30%)	66 (43.70%)	151	1.28
2010	111 (60.00%)	74 (40.00%)	185	1.5
2011	94 (55.62%)	75 (44.38%)	169	1.25
2012	93 (61.59%)	58 (38.41%)	151	1.60

* PB/MB: Ratio between paucibacillary and multibacillary.

Table 4 shows that the most prevalent clinical classification in almost every year was tuberculoid, followed by borderline, indeterminate and virchowian.

**Table 4:** Clinical classification of patients with leprosy in the city of Juazeiro-BA, between 2002 and 2012

	Clinical classification				
Year	Classification undetermined	Tuberculoid	Borderline	Virchowian	Total
2002	13 (5.81%)	117 (52.23%)	76 (33.93%)	18 (8.03%)	224
2003	23 (12.36%)	88 (47.31%)	62 (33.34%)	13 (6.99%)	186
2004	29 (15.27%)	76 (40.00%)	64 (33.68%)	21 (11.05%)	190
2005	14 (21.54%)	74 (48.38%)	48 (31.37%)	17 (11.11%)	153
2006	16 (11.60%)	71 (51.45%)	38 (27.54%)	13 (9.41%)	138
2007	13 (7.48%)	88 (50.57%)	49 (28.16%)	24 (13.79%)	174
2008	20 (12.74%)	61 (38.85%)	63 (40.13%)	13 (8.28%)	157
2009	24 (15.90%)	61 (40.40%)	52 (34.43%)	14 (9.27%)	151
2010	29 (15.67%)	82 (44.32%)	60 (32.43%)	14 (7.58%)	185
2011	34 (20.12%)	60 (35.51%)	62 (36.69%)	13 (7.68%)	169
2012	21 (13.91%)	72 (47.69%)	47 (31.12%)	11 (7.28%)	151

Table 5 presents the distribution of cases according to operational class, clinical form and sex. We can observe that borderline (35.72%) and virchowian (14.76%) forms were more prevalent in men (p <0.0001), which justifies a higher level of grade I (18.57%) and II (5.75%) disability also among men (p <0.0001).

**Table 5:** Association between clinical aspects of leprosy and gender in the city of Juazeiro-BA, between 2002 and 2012

Variables	Gender			
	Male	Female	P	OR (95% CI)
**Operational classification**				
Paucibacillary	446 (48.43%)	660 (66.33%)	<0.0001	2.09 (1.74-2.52)
Multibacillary	475 (51.57%)	335 (33.67%)		
				
**Clinical form**				
Tuberculoid	345 (37.46%)	505 (50.75%)	----	Reference
Indeterminate	92 (9.99%)	144 (14.47%)	0.6564	1.06 (0.79-1.43)
Borderline	329 (35.72%)	329 (35.72%)	<0.0001	1.64 (1.33-2.03)
Virchowian	136 (14.76%)	34 (3.42%)	<0.0001	5.85 (3.92-8.73)
Not informed	19 (2.07%)	20 (2.01%)		
				
**Number of lesions**				
< 5	519 (56.35%)	745 (74.88%)	<0.0001	2.30 (1.90-2.80)
≥ 5	402 (43.65%)	250 (25.12%)		
				
**Disability rate**				
Grade 0	639 (69.38%)	783 (78.70%)	----	Reference
Grade 1	171 (18.57%)	121 (12.16%)	<0.0001	1.73 (1.34-2.23)
Grade 2	53 (5.75%)	21 (2.11%)	<0.0001	3.09 (1.84-5.18)
Not informed	58 (6.30%)	70 (7.03%)		

Table 5 also shows that 402 men (43.65%) had >5 lesions, whereas the same quantity of lesions were observed in only 250 women (25.12%). Among individuals who developed <5 lesions, 519 were men (56.35%) and 745 were women (74.88%) (p <0.0001).

## DISCUSSION

The incidence rate in the city of Juazeiro was superior to those of the State of Bahia in the past few years. In 2010 the State presented a incidence rate of 19.21 per 100,000 inhabitants, and Juazeiro presented a rate of 94.97 per 100,000 inhabitants; that was also higher than the value found in the Northeast region in the same year, which was 27.73 per 100,000 inhabitants. These data shows that the disease is a major public health problem in the city, and that Juazeiro is far from achieving the leprosy elimination goal in the country set by the Ministry of Health.^10^

Thus, the city of Juazeiro can be considered hyperendemic by the classification parameters for leprosy in Brazil, presenting a ratio higher than 40.00 per 100,000 inhabitants.^10^

In most years, the incidence rate among women was higher than among men, being in conformity with the results found in the study of Prata, Bohland and Vinhas, where women accounted for 50.7% of leprosy cases diagnosed in Aracaju.^11^

The highest incidence of leprosy in women may be due to their habit of seeking medical care more often, while men usually seek medical care only when they present more severe symptoms and/or when they already have some disability.^11^

This result disagrees with results from other studies, such as Santos-Filho, where men represented 51.96% of leprosy cases in the city of Irecê-BA, and Silva-Sobrinho, Mathias and Lincoln, where men accounted for most cases of leprosy diagnosed in Paraná, representing 73.7% of cases.^12,13^

Results found in the most affected age groups have confirmed data from the literature, which demonstrate that leprosy is considered a disease of adults, because of the long incubation period. But children are also susceptible, particularly in endemic areas, and this is an important indicator for determining the transmission level.^14^

In a study performed in Irecê-BA, age groups with the highest expression were 35-49 years (33.3%), 50-64 years (31.9%), and 20-34 years (16.7 %)12. The results found in the most prevalent age groups can be attributed, partially, by the greater social mobility that individuals in these age intervals are exposed to, allowing contact with several people in several places, thus facilitating the contamination by *Mycobacterium leprae*.^15^

Another literature data estimates that the diagnosis of leprosy occurs on a mean age of 42 years. Thus, it seems that the disease usually manifests itself several years after contamination, taking into consideration the long incubation period of the disease. Since having a family member infected is a risk factor, it is recommended new assessments of intra-house-hold contacts, even years after the family member has completed the treatment regimen.^16^

Results showed the incidence of leprosy in children under 15 years with higher values than those found in the literature. In the city of Dourados-MS, this age group accounted for 2.5% of all cases, and in the city of Montes Claros-MG, between 2001 and 2009, boys accounted for 2.5% and girls for 3.4% of total cases.^2,17^ The incidence in this age group demonstrates early exposure and persistence of disease transmission, representing an important element to assess the disease's magnitude.^18^

The incidence rate in brown individuals predominated in both sexes, representing more than half of all cases, which is consistent with other data found in the literature.^2,14,16^

This result can be related to the fact that in Brazilian Northeast, brown individuals have predominance over other races due to a high level of miscegenation.^19^ However, at the Southern region of Santa Catarina, the white population showed the highest number of leprosy cases due to the predominance of that race in the region.^20^

The most reported level of education among patients with leprosy was incomplete elementary school, followed by illiterate, corroborating other studies.^16,21^

In the city of Dourados-MS, only 19.7% of patients had more than 8 years of study and most patients (79.0%) studied up to 8 years; 37.0% were illiterate or had 4 years of schooling completed.^17^ Therefore, grade of knowledge, access to health services, understanding the guidelines for the treatment and prevention measures are linked to self-care capacity and to the number of years of study.^22,23^

Almost 90% of cases were concentrated in urban areas, as shown in table 1, especially in its peripheries. Brazilian metropolis account for about 50% of cases in the country. Therefore, this can be considered as an urban endemy and areas with the greatest number of cases usually have a low socioeconomic standard and high population density, according to the literature.^24-28^

The variables that were good predictors for the development of physical disability, according to the logistic regression, were age >45 years old, male gender and multibacillary classification, which is consistent with other findings in the literature.^2^

Therefore, we can consider that these variables can serve as a warning to health care professionals, drawing attention to the need for reducing the social vulnerability of these patients.^2^

Table 3 shows that in all the years studied there were more paucibacillary cases, according to the study of Borges.^17^ Despite the higher prevalence of paucibacillary individuals, the number of multibacillary individuals is high, becoming alarming because it affects, especially, an economically active age group, and because it has a major impact on disease transmission.

The most present clinical form between 2002 and 2012 was tuberculoid, followed by borderline, indeterminate and virchowian. The fact that the tuberculoid type was the most prevalent in the city is worrying because it is an important epidemiological indicator of the increasing trend of the disease to affect competent individuals.^29,30^

In contrast, some studies have found a higher proportion of multibacillary individuals. In the study by Santos-Filho (2012), the clinical form that stands out most was the borderline (14.7%), followed by tuberculoid (12.2%), virchowian (10.3%), and indeterminate (6.9%).^2,12,13,31,32^

A higher proportion of more severe forms of leprosy, borderline and virchowian, in men compared to women, is associated with a higher rate of disability also found in males. These data are similar to those observed in the study of Ribeiro-Júnior, Vieira and Caldeira, which assessed the epidemiological profile of leprosy in an endemic city in the North of Minas Gerais, as well as the study of Hinrichsen *et al* performed in the city of Recife-PE.^2,27^

Attention should be drawn to the low percentage of indeterminate form of the disease (9.99% in men and 14.47% in women), since this information may be related to a delay in diagnosis, possibly because primary healthcare centers fail in detecting cases in the early forms of the disease (Table 4).^33,34^

Leprosy would not be such an impacting disease if it were just a contagious skin disease. However, its predilection for the peripheral nerves resulting in disabilities and deformities, causes fear, prejudice and taboos about the disease.^35^

It is necessary to increase the diagnostic network to enable the early detection of the disease and thus reducing the number of physical disabilities.^12,31^ Neural damage is the main cause of disability, resulting in limitations of activities and social participation of people affected by the disease.^36^

## CONCLUSION

This study shows alarming results of the leprosy incidence rate in the city of Juazeiro-BA. Regarding the presented data, we can conclude that the incidence rate is decreasing over the studied years, but it is still far from the values considered low by the Ministry of Health. We observed that the most affected population were women, brown individuals, those with low level of education, living in the urban area, and those living in the urban area.

Results of logistic regression showed that there are greater chances of developing some disability in individuals >45 years, men, and those classified as multibacillary. The most severe forms of leprosy were detected, mostly in men, probably because they do not have the same care with health than women, only seeking health services when the disease is already causing a disability. This reinforces that the poor knowledge of the disease and the delay in diagnosis are key factors in the development of the most serious and disabling forms of the disease.

Due to lack of studies like this in Juazeiro, this research becomes relevant to better understand the magnitude of leprosy in the city, thereby, directing the control measures for this disease, which is one of the Ministry of Health targets.

Therefore, we should intensify efforts in the active search for new cases of the disease in the city, as well as increasing health education activities, which are of great importance. With a better knowledge of the disease by the population it is possible that existing cases can be diagnosed and treated in the early stage of the disease, so that there may be a break in the chain of transmission of leprosy in this city.
